# Preparation of In Situ Growth Multiscale β-Sialon Grain-Reinforced Al_2_O_3_-Based Composite Ceramic Tool Materials

**DOI:** 10.3390/ma16062333

**Published:** 2023-03-14

**Authors:** Jian Zhu, Yunna Xue, Xiaolan Bai, Xuehui Shen, Jianqun He, Yu Zhang, Anhai Li

**Affiliations:** 1 School of Mechanical and Automotive Engineering, Qilu University of Technology (Shandong Academy of Sciences), Jinan 250353, China; 2 Shandong Institute of Mechanical Design and Research, Jinan 250353, China; 3 Laser Institute, Qilu University of Technology (Shandong Academy of Sciences), Jinan, 250103, China; 4 Key Laboratory of High Efficiency and Clean Mechanical Manufacture of MOE, School of Mechanical Engineering, Shandong University, Jinan 250061, China

**Keywords:** ceramic composite, in situ growth, multiscale β-sialon grains, microstructure, mechanical properties

## Abstract

A kind of multiscale β-sialon grain-reinforced Al_2_O_3_ matrix composite ceramic tool material, named ASN, was prepared and studied. For the ASN, β-sialon (molecular formula: Si_4_Al_2_O_2_N_6_) was synthesized in situ by a hot-pressing and solid-solution reaction process. A total of six samples were prepared at varying sintering temperatures and holding times under vacuum conditions. The solid solution reaction mechanism of β-sialon, the phase composition, mechanical properties, microstructure, and strengthening and toughening mechanisms of the composite ASN were investigated. As a result, within the experimental parameters, an optimal ASN tool material was obtained under a pressure of 32 MPa and at a temperature of 1550 °C for 20 min. The tested mechanical properties of the optimal sample were as follows: flexural strength 997 ± 59 MPa, fracture toughness 6.4 ± 0.3 MPa·m^1/2^, Vickers hardness 18.2 ± 0.4 GPa, and relative density 98.1 ± 0.2%. According to crystal defect theory, the solid solution reaction mechanism of in-situ-synthesized β-sialon in an Al_2_O_3_ matrix involves a double mechanism of unequivalence (or hetero-valence) and interstitial filling. The multiscale β-sialon grains mainly consisted of four grains, which were elongated β-sialon grains with a diameter of 0.3–0.4 μm and an aspect ratio of 6–9, elongated β-sialon grains with a diameter of 70 nm and an aspect ratio of 10, β-sialon whiskers with a diameter of 0.2 μm and an aspect ratio of 12–15, and intragranular β-sialon whiskers with a diameter of 70 nm. The mechanical properties were improved due to strengthening and toughening mechanisms, such as mixed structure mode (intergranular and transgranular), elongated grain pullout, interface bonding, crack reflection, pinning, and bridging.

## 1. Introduction

In order to process difficult-to-cut materials commonly used in the automobile, aircraft and astronautic manufacturing industries (such as hardened steel, nickel-based and titanium alloys, etc.), advanced ceramic tools must have high mechanical and thermal properties [1,2,3,4,5,6]. For example, at ambient temperature, the flexural strength, fracture toughness, and Vickers hardness of Al_2_O_3_/TiC micro–nano-composite ceramic tool material were 916 MPa, 8.3 MPa·m^1/2^ and 18 Gpa, respectively, and still maintained a high flexural strength value (about 390 MPa) at 1200 °C. However, ceramic materials are limited by their inherently brittle nature, often resulting in catastrophic failure, restricting their application. The strength and fracture toughness of the composites can be greatly improved by adding elongated grains, whiskers or chopped fibers to the ceramic matrix, such as SiC whiskers and SiC chopped fibers toughening ZrB_2_-based ceramics [7], ZrB_2_ whiskers and elongated grains toughening ZrB_2_–ZrC ceramic tool materials [8,9], TaC whiskers toughening Al_2_O_3_-based composites [10], elongated β-Si_3_N_4_ grain-reinforced Al_2_O_3_-based composites [11], and elongated β-sialon grain-toughened WC-based cermets [12], etc. However, directly adding whiskers and elongated grains presents difficulties for dispersion and compaction in the matrix. Therefore, in situ synthesis technology is still an effective way to overcome these shortcomings.

β-sialon ceramics have been widely researched for their good sintering properties, high fracture toughness and typical rod-like structure [10,13]. β-sialon, as a promising reinforcing phase, has attracted much attention due to its excellent mechanical properties, stable chemical properties and good thermal properties [14,15,16]. β-sialon is regarded as a solid solution isostructural to β-Si_3_N_4_ obtained by the solid solution reaction of Si_3_N_4_ and Al_2_O_3_ at high temperature. Its chemical formula is Si_6−z_Al_z_O_z_N_8−z_ (the z value range is 0~4.2), where z represents the number of Si and N atoms replaced by Al and O, respectively. With increase in z value, the number of Al and O atoms entering the β-Si_3_N_4_ lattice was observed to increase, and the cell size also increased. However, β-sialon still retained the hexagonal system of β-Si_3_N_4_ and had the characteristics of anisotropic grain growth along the c-axis [14,17,18,19]. Obviously, β-sialon is formed by replacing the Si-N bond with the Al-O bond, and it is difficult to obtain high density without a grain boundary phase during the sintering of β-sialon ceramics. Hence, pressure-assisted sintering techniques and the addition of sintering oxide additives have been applied to prepare fully compacted bodies, such as hot pressing (HP) [20], hot isostatic pressing (HIP) [21], spark plasma sintering (SPS) [22], and use of oxide sintering aids (namely Al_2_O_3_, MgO and rare earth oxides) [13,23,24,25]. In particular, some rare earth oxides (such as Y_2_O_3_, Yb_2_O_3_, etc.) did not dissolve into β-sialon during sintering, but existed on the grain boundary as a crystalline phase. Moreover, these rare earth oxides cannot only reduce the grain boundary energy and promote sintering between grains, but can also promote the development of elongated β-sialon [24,25,26]. Of these oxides, Y_2_O_3_ is generally considered to be the preferred additive for preparing β-sialon ceramics.

According to the Si_3_N_4_-Al_2_O_3_-SiO_2_-AlN system phase diagram produced by K.H. Jack [17], the synthesis of β-sialon comprises three reaction pathways, namely

Si_3_N_4_ + AlN + Al_2_O_3_→Si_6-z_Al_z_O_z_N_8-z_ (1)

Si_3_N_4_ + AlN + SiO_2_→Si_6-z_Al_z_O_z_N_8-z_ (2)

Si_3_N_4_ + Al_2_O_3_ + SiO_2_→Si_6-z_Al_z_O_z_N_8-z_ (3)

In the above three equations, the synthesis pathways described by reactions (1) and (2) have been adopted by some researchers [16,27,28]. The intermediate transition phase AlN was generated by adding Al_2_O_3_ as an oxygen source to an Si_3_N_4_ matrix, and then β-sialon ceramics were prepared by a high temperature nitriding process in a nitrogen atmosphere. The pathway described by reaction (3) was also adopted by some scholars [29,30,31]; β-sialon ceramics were prepared by intentionally adding various oxide sintering aids in the sintering process to form a low melting point liquid phase with high saturated nitrogen; the nitrogenous saturated oxide melt was considered to trigger the precipitation of β-sialon. 

To date, Al_2_O_3_/β-sialon ceramic tool materials prepared by in situ synthesis technology have rarely been reported. In our present work, in situ synthesis of β-sialon ceramics as a reinforcing phase in the Al_2_O_3_ matrix was undertaken by hot pressing and solid solution reaction sintering. Based on crystal defect theory, the solid solution reaction mechanism of β-sialon was investigated in the Al_2_O_3_ matrix. In addition, the phase composition, mechanical properties, microstructure, and the toughening and strengthening mechanisms of tool materials, were also investigated. The preparation process and room temperature mechanical properties of ASN and partial Al_2_O_3_-based ceramic tool materials are compared in Table 1.

## 2. Experimental Procedures

### 2.1. Materials and Preparation Procedures

The raw material powders used were commercially available α-Al_2_O_3_ powders with an average grain size of 0.5 µm (purity: 99.99%, Zibo, China), and Si_3_N_4_ powders with an average grain size of 0.5 µm (purity: 99.9%, Shanghai, China). The Si_3_N_4_ powders contained an α phase with a mass fraction of above 92%. Our previous results indicated that adding 1.5 wt.% Y_2_O_3_ to Al_2_O_3_-Si_3_N_4_ composite powders was an effective way to improve densification and interfacial bonding strength [11]. In addition, Lee et al. found that the z values for β-sialon were independent of the amount of liquid [32]. Therefore, in this study, chemically pure Y_2_O_3_ was selected as a sintering additive and the previous Al_2_O_3_-25 wt.% Si_3_N_4_-1.5 wt.% Y_2_O_3_ mixed powders were also used. The ternary Al_2_O_3_-Si_3_N_4_-Y_2_O_3_ mixed powders were ball-milled with ethanol for 48 h in a polyethylene jar and were then dried in a vacuum dry-type evaporator (Model ZK-82A, Shanghai, China). After that, the dried powders were sieved through a 120-mesh sieve for further use. The dried powders were placed into a cylindrical graphite die which was coated with a thin layer of hexagonal boron nitride (h-BN) on the inner wall to demold easily. The previous research results showed that no sialon phase was generated at the sintering temperature of 1450–1500 °C. Therefore, in this investigation, the composite ceramic tool materials were prepared via hot-pressing technology in vacuum at temperatures above 1500 °C for 10–30 min under a uniaxial pressure of 32 MPa.

### 2.2. Characterization

The sintered compacts were cut (J5060C-1-type inner-circular cutter), and ground (UNIPOL-1502 precision lapping machine) into bars with dimensions of 3 mm × 4 mm × 40 mm. Then, the bars’ edges were chamfered with dimensions of about 0.1 mm × 0.1 mm and ground to eliminate machining flaws on the edges. Finally, the bars were polished with a diamond spray. The flexural strength was measured using a three-point bending tester (Model WD-10, Jinan, China) with a span of 20 mm and a loading velocity of 0.5 mm/min. The Vickers hardness was measured on the polished surface using a Vickers diamond pyramid indenter (Model 120, Hebei, China) with a load of 196 N and a holding time of 15 s. The fracture toughness measurement of the composites was determined by the Vickers indentation method proposed by Evans and Charles [33]. At least six specimens were tested for each mechanical property. The density of each specimen was measured using a solid density tester (Model DH-120, Kawasaki, Japan) by the Archimedes immersion technique with distilled water as the immersion medium; three specimens were tested for each experimental condition. Phase identification was carried out by X-ray diffraction analysis (XRD, RAX-10A-X, Hitachi, Tokyo, Japan) with copper Kα radiation. A scanning electron microscope (SEM, SUPRA-55, ZEISS, Jena, Germany) equipped with an energy dispersive spectrometer (EDS, PV9900 Philips, Amsterdam, The Netherlands) was used to observe the microstructure and chemical constituent of the composites. The polished surfaces for the SEM observations were chemically etched in molten NaOH at 400 °C for 60 s. Since the composite under investigation was non-conducting, the specimen surfaces were sprayed with a thin layer of gold for clear observation during SEM examination.

## 3. Results and Discussion

### 3.1. Phase Composition, Mechanical Properties and Microstructure

#### 3.1.1. Effect of Sintering Temperature 

**Figure 1:**
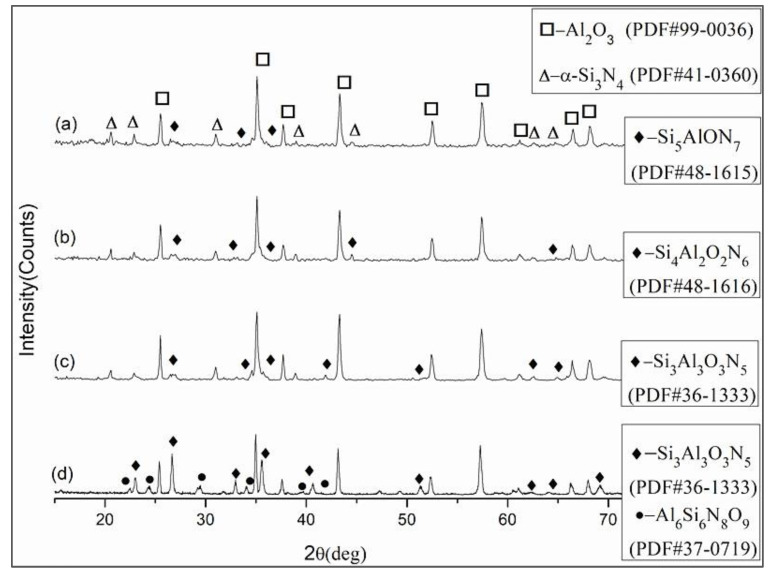
XRD patterns of the composite tested at (**a**) 1525 °C, (**b**) 1550 °C, (**c**) 1575 °C, and (**d**) 1600 °C.

The composite ceramic material prepared by hot pressing and solid solution reaction sintering of the ternary Al_2_O_3_-25 wt.% Si_3_N_4_-1.5 wt.% Y_2_O_3_ mixed powders was named ASN. The XRD patterns of ASN samples prepared at 1525–1600 °C for 20 min under 32 MPa are shown in Figure 1. It can be seen from Figure 1 that three solid solutions with different molecular formulas were detected in the ASN ceramic materials; the three chemical molecular formulas were Si_5_AlON_7_ (lattice parameters: a = b = 7.635 Å, c = 2.934 Å; density ρ = 3.156 × 10^3^ kg/m^3^), Si_4_Al_2_O_2_N_6_ (lattice parameters: a = b = 7.666 Å, c = 2.960 Å; density ρ = 3.112 × 10^3^ kg/m^3^) and Si_3_Al_3_O_3_N_5_ (lattice parameters: a = b = 7.678 Å c = 2.977 Å; density ρ = 3.095 × 10^3^ kg/m^3^) at 1525 °C, 1550 °C and 1575–1600 °C, respectively. Si_5_AlON_7_, Si_4_Al_2_O_2_N_6_ and Si_3_Al_3_O_3_N_5_ were consistent with β-sialon’s formula Si_6−z_Al_z_O_z_N_8−z_, and correspondingly, the z values were 1, 2, and 3, indicating that substitution of Al-O for Si-N increased with increase in sintering temperature. Yttria, as a conventional sintering additive has been proved to be an effective means of promoting densification [34]; however, no Y_2_O_3_ was detected due to its low mass fraction. Furthermore, the amount of β-sialon was estimated quantitatively by the ratio of the diffractive intensities of α-Si_3_N_4_ and β-sialon peaks observed from the XRD spectrum in Figure 1. The results revealed that at 1525 °C, 1550 °C, 1575 °C, and 1600 °C, about 28%, 36%, 38%, and 100% of α-Si3N4 mass fraction underwent the solid solution reaction with Al_2_O_3_ after the phase transition, respectively. The content of β-sialon was less at 1525 °C than at 1550–1600 °C, and the contents of Si_4_Al_2_O_2_N_6_ and Si_3_Al_3_O_3_N_5_ in the ASN composite materials were nearly equivalent at 1550 °C and 1575 °C, respectively. It can be seen from Figure 1a–c that the ASN composite ceramic materials were mainly composed of Al_2_O_3_, α-Si_3_N_4_ and β-sialon at 1550–1575 °C. However, it can be seen in Figure 1d that, in addition to β-sialon (chemical formula: Si_3_Al_3_O_3_N_5_), an impurity phase Al_6_Si_6_N_8_O_9_ was generated in ASN at 1600 °C, which was very unfavorable to the preparation of ceramic tool materials.

**Figure 2:**
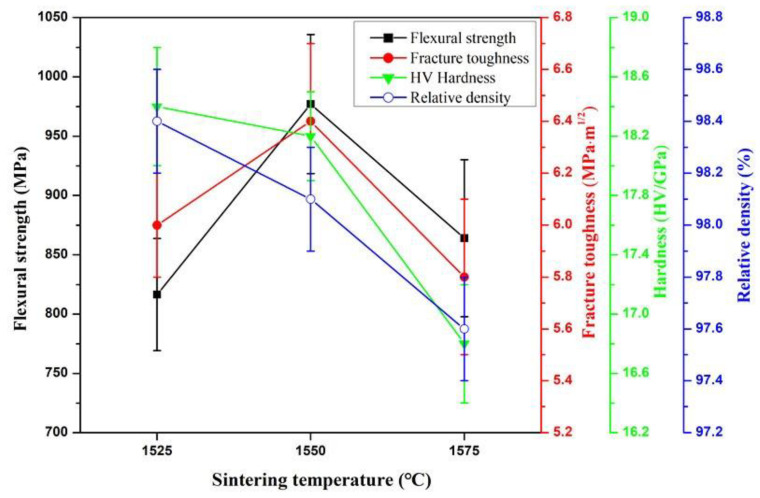
Effects of sintering temperature on flexural strength, fracture toughness, hardness and relative density of ASN sintered under 32 MPa for 20 min.

The flexural strength, fracture toughness, Vickers hardness and relative density of ASN sintered under 32 MPa for 20 min at different sintering temperatures (1525 °C, 1550 °C and 1575 °C) are shown in Figure 2. As shown in the figure, with increase in sintering temperature from 1525 °C to 1575 °C, the flexural strength and fracture toughness of ASN first increased and then decreased, reaching a maximum at 1550 °C, while the Vickers hardness and relative density decreased with increase in temperature. Compared with the results shown in Figure 2, ASN samples showed good comprehensive mechanical properties and high relative density at 1550 °C. 

The changes in the mechanical properties and relative density of the ASN sample at different sintering temperatures were related to the composition and quantity of β-sialon. Tanaka et al. [35] indicated that the properties of β-sialon were directly related to the z value. As the value of z increased, β-sialon cell size increased, the strength of atomic covalent bonds decreased, and the lattice softened, resulting in reduction in its elastic modulus, Vickers hardness, density and flexural strength; in particular, the elastic modulus and Vickers hardness decreased almost linearly. As shown in Figure 1a–c, with increase in temperature, the z value of Si-N replaced by Al-O increased, with z = 1, z = 2 and z = 3 at 1525 °C, 1550 °C and 1575 °C, respectively. Compared with the results shown in Figure 2, due to β-sialon (i.e., Si_5_AlON_7_), a small z value at 1525 °C occurred, and the Vickers hardness and relative density of ASN were higher than those at 1550 °C and 1575 °C. However, the low content of Si_5_AlON_7_ limited its toughening and strengthening effects on ASN. In addition, although the amounts of Si_4_Al_2_O_2_N_6_ (at 1550 °C) and Si_3_Al_3_O_3_N_5_ (at 1575 °C) were approximately equivalent in ASN, according to the results of Tanaka et al., Si_4_Al_2_O_2_N_6_ ceramic tool materials have higher flexural strength and fracture toughness than Si_3_Al_3_O_3_N_5_, as shown in Figure 2. Therefore, β-sialon prepared at 1550 °C had a good reinforcement effect on the ASN composite ceramics.

**Figure 3:**
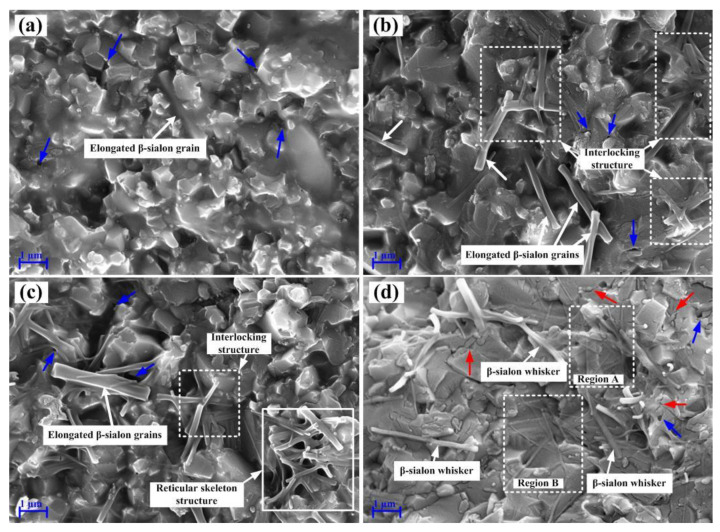
SEM micrographs on fracture surfaces of ASN samples sintered under 32 MPa for 20min at (**a**) 1525 °C, (**b**) 1550 °C, (**c**) 1575 °C, and (**d**) β-sialon whiskers in ASN sample sintered at 1550 °C.

Furthermore, the mechanical properties and relative density of ASN also depend on microstructure. SEM micrographs of the fracture surfaces of ASN samples sintered at different temperatures are shown in Figure 3. As shown in Figure 3, a certain number of pores (denoted by blue arrows) were distributed not only at the grain boundaries, but also in the grains. The pores can lead to poor densification of ASN. In addition, pores can also decrease the loaded cross-sectional area of ASN samples, resulting in stress concentration and, thus, impairment of the mechanical properties of ASN. It can also be seen from Figure 3a–c that, when the sintering temperature increased from 1525 °C to 1575 °C, many elongated β-sialon grains (denoted by white arrows) with different aspect ratios were synthesized in situ in the Al_2_O_3_ matrix; the aspect ratio and diameter were about 6–9 and 0.3–0.4 μm, respectively. As shown in Figure 3d, it was also observed that β-sialon whiskers with an aspect ratio of 12–15 and a diameter of about 0.2 μm (denoted by white arrows), β-sialon whiskers with a nano diameter (region A and region B indicated by the quadrate dotted box), and their interlocking structures, were produced at 1550 °C for 20 min. It was predicted that the flexural strength and fracture toughness of the dense ceramics could be increased by the addition of a moderate quantity of the elongated grains or whiskers [36]. As a result, as shown in Figure 2, the elongated particles and whiskers of β-sialon were able to effectively improve the strength and toughness of ASN tool materials sintered at 1550 °C. However, the elongated β-sialon grains and β-sialon whiskers were irregularly distributed in the Al_2_O_3_ matrix, as shown in Figure 3b,d, which led to the inhomogeneous microstructure and limited the further improvement of the mechanical properties of ASN. In addition, some micro-cracks (denoted by red arrows) were observed at the grain boundaries, as shown in Figure 3d. According to the Griffith theory of fracture strength, for the micro-cracks at the grain boundaries, when the load was applied to ASN, these cracks did not only cause the stress concentration and weaken the grain-boundary strength, but also propagated easily, which impaired the flexural strength of the ASN ceramic tool materials. 

It is shown in Figure 3b,c that the interlocking structures (indicated by the white quadrate dotted box) and the reticular skeleton structure (indicated by the white quadrate box) of elongated β-sialon grains were present in the ASN composites. The two network structures of β-sialon grains have been interpreted to result from a solution-precipitation mechanism caused by the low-melting point liquid phase formed at the sintering temperature [37]. Although the interlocking and skeleton structures can cause ASN tool materials to have a high bearing capacity, their random and inhomogeneous distribution limited the improvement of strength and toughness of ASN. In particular, the large-sized pores in the skeleton structure caused the density and hardness of the tool material to decrease sharply, resulting in low flexural strength and fracture toughness at 1575 °C.

#### 3.1.2. Effect of Sintering Time 

**Figure 4:**
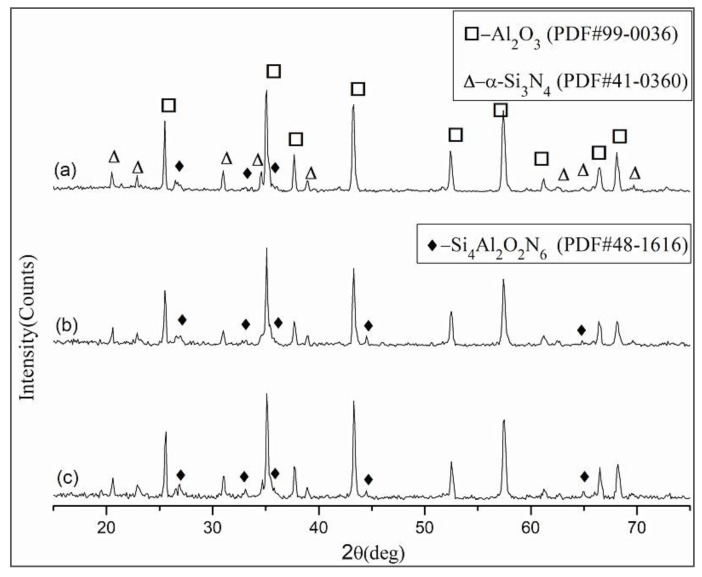
XRD patterns of ASN sintered at 1550 °C for (**a**) 10 min, (**b**) 20 min, and (**c**) 30 min.

The XRD patterns of ASN sintered at 1550 °C for 10 min, 20 min, and 30 min. are shown in Figure 4. It can be seen that the composite ceramic materials of different sintering times were composed of Al_2_O_3_, α-Si_3_N_4_ and β-sialon (chemical formula: Si_4_Al_2_O_2_N_6_). No evidence of any new phases was detected in the XRD spectrum, indicating that the number of Al-O substituted Si-N did not increase with increase in the sintering time at 1550 °C; that is, the value of z was still 2. However, comparing the diffractive intensities of the β-sialon peaks, the quantity of β-sialon with the molecular formula Si_4_Al_2_O_2_N_6_ increased with increase in sintering time. Yttria was still not detected due to its low mass fraction.

**Figure 5:**
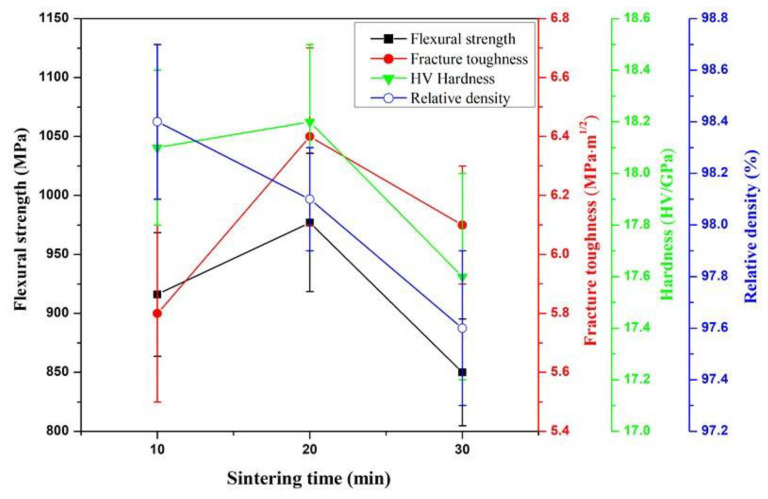
Effects of sintering time on flexural strength, fracture toughness, hardness and relative density of ASN sintered at 1550 °C under 32 MPa.

The effects of the sintering time on the mechanical properties and relative density of ASN sintered at 1550 °C are shown in Figure 5. It can be seen from Figure 5 that the mechanical properties of ASN first increased and then decreased as the sintering time increased from 10 min to 30 min, reaching a maximum at 20 min. However, the relative density gradually decreased with increase in sintering time. As shown in the comparative results in Figure 5, when the sintering time was 20 min, the ASN sample possessed optimal comprehensive mechanical properties and higher relative density; the flexural strength, fracture toughness, Vickers hardness and relative density were 997 ± 59 MPa, 6.4 ± 0.3 MPa·m^1/2^, 18.2 ± 0.4 GPa and 98.1 ± 0.2%, respectively.

**Figure 6:**
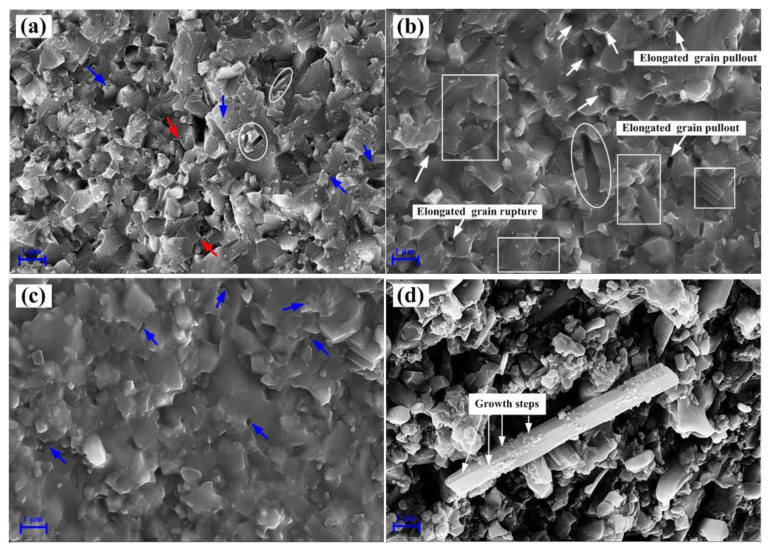
SEM micrographs on fracture surfaces of ASN samples sintered under 32 MPa at 1550 °C for (**a**) 10 min, (**b**) 20 min, (**c**) 30 min, and (**d**) abnormally grown β-sialon grain for 30 min.

SEM micrographs on the fracture surfaces of ASN at 1550 °C for the different sintering times are shown in Figure 6. As shown in Figure 6a, when the sintering time was 10min, some pores (denoted by the blue arrows) and micro-cracks (denoted by the red arrows) appeared at the grain boundaries, which reduced the mechanical properties and relative density of ASN. Although no obvious elongated β-sialon grains are evident in Figure 6a, cleavage planes of the elongated grains being pulled out (indicated by the ellipse box) were found, and the aspect ratio and diameter were about 4–6 and 0.2 μm, respectively. It is speculated that β-sialon grains were stunted along the c-axis direction in the short sintering time, and that, thus, the improvement in mechanical properties was limited. As the sintering time was increased to 20min, compared with the microstructure shown in Figure 6a, the microstructure shown in Figure 6b became dense and homogeneous, and enhanced the mechanical properties. When the sintering time was up to 30 min, it can be seen from Figure 6c that the grain size was relatively coarse, the grain boundary was unclear, and some pores (denoted by the blue arrows) were distributed at the grain boundary and in coarsened grains, which resulted in the mechanical properties and the relative density of the ASN ceramic material decreasing (in line with the results shown in Figure 4). In particular, when the holding time was 30 min, abnormally grown elongated β-sialon grain with an aspect ratio of about 9 and a diameter of about 1 μm were also observed, as shown in Figure 6d. This indicated that the anisotropic β-sialon grains coarsened quickly and that the microstructure became inhomogeneous with increase in the sintering time. Moreover, growth steps along the length direction were also observed from the elongated β-sialon grains with straight and hexahedral structures, as shown in Figure 6d. The emergent growth steps of the β-sialon grains have been understood in terms of the solid–liquid–solid mechanism and the anisotropic Ostwald ripening growth mechanism, similar to the growth of β-Si_3_N_4_ whiskers [36,38]. However, the development of the β-sialon grains in ASN clearly still conformed to the solution-precipitation mechanism [39].

**Figure 7:**
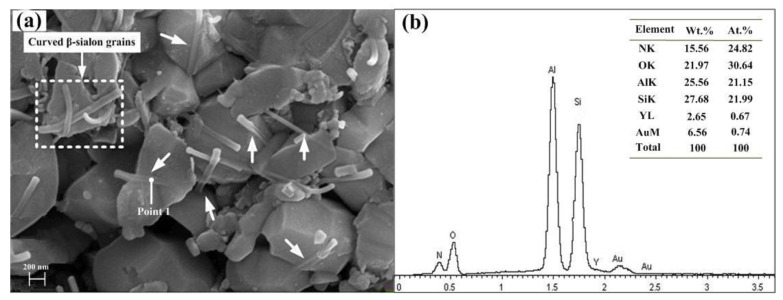
(**a**) SEM micrograph on etched surface of ASN sample sintered at 1550 °C for 20 min, and (**b**) EDS results of point 1 in (**a**).

In order to further investigate the microstructure of ASN sintered at 1550 °C for 20 min, the ASN sample shown in Figure 6b was chemically etched. An SEM image of the etched surface of the ASN sample and the chemical constituents detected by EDS in point 1 are shown in Figure 7. As can be seen from Figure 7a, on the one hand, some straight β-sialon whiskers with a diameter of about 70 nm (denoted by the white arrows) were embedded in large particles with a diameter of about 1 μm to form an intragranular structure. The β-sialon grains were not completely embedded in the Al_2_O_3_ matrix grains but were partially exposed. On the other hand, some elongated β-sialon grains, with the same nano diameter and an aspect ratio of 10, were not enfolded by the large particles, forming an intergranular structure in which some β-sialon grains were curved and intertwined with each other (indicated by the quadrate dotted box in Figure 7a). The chemical constituents detected by EDS in point 1, as shown in Figure 7b, were Si, Al, O N and Y, which revealed that the production of intragranular β-sialon was related to the liquid phase during the sintering process as a result of the presence of Y element in the EDS results. A liquid phase with low melting point was formed easily among Al_2_O_3_, Y_2_O_3_ and SiO_2_ present on the surface of the Si_3_N_4_ particles under the hot-pressing condition. Compared with α-Si_3_N_4_, β-Si_3_N_4_ and β-sialon have similar nucleation stacking sequences and less different lattice parameters. In terms of energy, β-Si_3_N_4_ should be the preferred nucleation site for β-sialon due to a smaller interface energy and strain energy during the solid solution reaction. Therefore, β-sialon should also be a solid solution reaction product of Al_2_O_3_ and β-Si_3_N_4_. 

**Figure 8:**
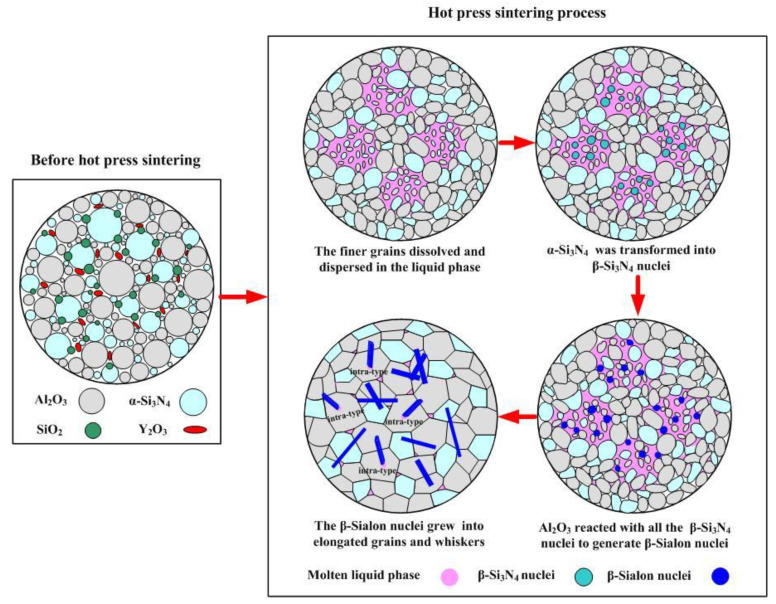
Schematic diagram of the formation of β-sialon’s intergranular and intragranular structures under a dissolution–transport–precipitation mechanism.

As shown in Figure 8, when the nitrogenous saturated oxide melt Al_2_O_3_-Y_2_O_3_-SiO_2_ was formed under high temperature sintering, the finer Al_2_O_3_ and α-Si_3_N_4_ grains with the higher surface energy were preferentially dissolved and rapidly dispersed in the liquid phase. Once the phase transition temperature was reached, some finer α-Si_3_N_4_ grains dissolved in the liquid phase were transformed into β-Si_3_N_4_ nuclei. Then, under the hot-pressing condition, the finer Al_2_O_3_ grains with the higher atomic diffusion rate and β-Si_3_N_4_ nuclei with higher activation energy underwent a solid solution reaction to form β-sialon crystal nuclei, with a part of the β-sialon crystal nuclei enfolded by the finer Al_2_O_3_ grains. As the sintering continued, the finer Al_2_O_3_ grains gathered around the β-sialon nuclei were rearranged and grew into large particles, while the β-sialon crystal nuclei also grew into elongated grains or whiskers. After the sintering was complete, the elongated β-sialon grains precipitated from the liquid phase; part of the β-sialon grains were trapped inside the large Al_2_O_3_ particles (about 1 μm in diameter) to form an intra-type microstructure, and the other part of the β-sialon grains remained at the Al_2_O_3_-α-Si_3_N_4_ boundaries to form an inter-type microstructure. At the same time, the liquid phase was finally retained at the two-grain boundaries [11]. Therefore, an intra-type β-sialon whiskers structure in the ASN composite ceramic tool materials was generated through a dissolution–transport–precipitation mechanism.

### 3.2. Solid Solution Reaction Mechanism of β-Sialon Formation

It can be seen from Figure 1 that, unlike in the silicon nitride matrix, in the aluminum oxide matrix, the solid solution reaction of Al_2_O_3_ and Si_3_N_4_ was directly generated in a narrow temperature interval (1525–1575 °C) without other intermediate transition phases (such as AlN) and excess β-Si_3_N_4_ being produced, which indicated that the amount of solute and solvent involved in the solid solution reaction was appropriate in this study. It is speculated that the solid solution reaction mechanism of in-situ-synthesized β-sialon in the Al_2_O_3_ matrix may be different from that in the Si_3_N_4_ matrix. Due to the low content of Y_2_O_3_ and the limited amount of SiO_2_ originated from the surface of Si_3_N_4_ particles, the Al_2_O_3_-Y_2_O_3_-SiO_2_ system liquid phase that promoted the transformation from α-Si_3_N_4_ to β-Si_3_N_4_ was also less, resulting in a reduced amount of β-Si_3_N_4_ [40,41]. A small amount of β-Si_3_N_4_ participated in the solid solution reaction. The number of Si and N atoms replaced by Al and O was also decreased accordingly, thus limiting the amount of β-sialon. Therefore, it is speculated that the solid solution reaction process in this study was more consistent with the above Equation (3); namely, β-sialons may be in situ synthesized in the Si_3_N_4_-Al_2_O_3_-SiO_2_ ternary reaction system. 

It is well known that the high-temperature solid solution reaction between crystals is related to the crystal defects of reactants. With increase in temperature, some atoms in the crystal always deviate from their equilibrium positions, causing lattice distortion and crystal defects. According to crystal defect theory, the ionic compound Al_2_O_3_ easily forms Schottky vacancy defects, while the covalent compound Si_3_N_4_ containing SiO_2_ on the surface (represented by Si_3_N_4_•SiO_2_) easily forms Frenkel defects. The crystal defects are described by Kroger–Vink notation as follows:

(4) $$Al_{2}O_{3}\to 2V_{Al}^{\prime \prime \prime }+3V_{O}^{••}$$

(5) $$Si_{3}N_{4}\cdot SiO_{2}\to 2Si_{i}^{••••}+2V_{Si}^{{}^{\prime }{}^{\prime }{}^{\prime }{}^{\prime }}+2N_{i}^{\prime \prime \prime }+2V_{N}^{•••}+O_{i}^{\prime \prime }+V_{O}^{••}$$

It can be seen from the description of Equation (4) that cationic Al^3+^ and anion O^2−^ deviate from their original normal positions in alumina crystal, at the same time, resulting in vacancy pairs with corresponding negative and positive charges (namely $V_{Al}^{\prime \prime \prime }$ and $V_{O}^{••}$). It can be seen from the description of Equation (5) that Si atoms, N atoms and O atoms deviate from their normal positions and enter the lattice gaps in the Si_3_N_4_•SiO_2_ crystal, resulting in defect pairs composed of corresponding vacancies and interstitials (namely, $V_{Si}^{{}^{\prime }{}^{\prime }{}^{\prime }{}^{\prime }}$ and $Si_{i}^{••••}$, $V_{N}^{•••}$ and $N_{i}^{\prime \prime \prime }$, $V_{O}^{••}$ and $O_{i}^{\prime \prime }$). Obviously, the crystal defect is the charge defect, that is, the distortion of the electric potential field around the crystal, and the solid solution reaction occurs because of the charge imbalance. Therefore, the solid solution reaction between Al_2_O_3_ and Si_3_N_4_ is the process of charge rebalancing after the charge defects are generated in Al_2_O_3_ and Si_3_N_4_, respectively. 

In this study, α-Si_3_N_4_ was transformed into β-Si_3_N_4_ in the liquid phase of Al_2_O3-Y_2_O_3_-SiO_2_ system at high temperature; meanwhile, Al_2_O_3_ and β-Si_3_N_4_ nuclei were prone to solid solution reaction due to the existence of charge defects around them. When the solid solution reaction occurred, Al^3+^ and O^2−^ replaced Si^4+^ and N^3−^; that is, low-priced cations and anions replaced high-priced cations and anions, and, thus, there were differences in the electricity prices. This dual replacement of different prices must create an imbalance in the electricity price. In order to maintain the electric neutrality of the β-sialon structure, the electricity prices must be compensated. Usually, the compensation methods of electricity price balance include the ion vacancy mechanism and the ion interstitial filling mechanism. As β-Si_3_N_4_ is a covalently bonded crystal, there are few vacancies in the crystal, but there are interstitials in the crystal. Therefore, under the condition of hot pressing, when Al_2_O_3_ and β-Si_3_N_4_ dissolved in the liquid phase, the Al_2_O_3_-Y_2_O_3_-SiO_2_ system underwent a solid solution reaction, Al^3+^ and O^2−^ diffused into the interstitial position of the β-Si_3_N_4_ crystal, and the interstitial replacement occurred to achieve equilibrium of the electricity price. 

Furthermore, the β-sialon formula was Si_4_Al_2_O_2_N_6_ and the number of Al-O replacing Si-N was 2 in this investigation. According to crystal defect theory, the principle of a solid solution reaction is that the charge, mass and atomic number involved in the reaction are mutually conserved. Thus, the equation of the solid solution reaction by adding silicon nitride into an alumina matrix to generate β-sialon is as follows:

(6) $$3Al_{2}O_{3}\overset{\;Si_{3}N_{4}•SiO_{2}\;}{\to }4A{l^{\prime }}_{Si}+4O_{N}^{•}+2O_{o}^{x}+2Al_{i}^{•••}+3{O^{\prime \prime }}_{i}$$

where, $Al_{Si}^{\prime }$, $O_{N}^{•}$, $Al_{i}^{•••}$, $O_{i}^{\prime \prime }$ and $O_{o}^{x}$ are the Kroger–Vink symbols of Si and N atoms replaced by Al and O atoms. $Al_{Si}^{\prime }$ indicates that Al^3+^ replaces Si^4+^ at the Si site, while adding a negative charge. $O_{N}^{•}$ indicates that O^2-^ replaces N^3−^ at the N site, while adding a positive charge. $Al_{i}^{•••}$ indicates that Al^3+^ occupies the interstitial site of Si_3_N_4_ crystal, adding three positive charges at the same time. $O_{i}^{\prime \prime }$ indicates O^2−^ occupies the interstitial site of Si_3_N_4_ crystal, adding two negative charges at the same time. $O_{o}^{x}$ indicates that O^2−^ in Al_2_O_3_ replaces the O interstitial site in SiO_2_ and the charge remains neutral. Among these, the presence of SiO_2_ plays the role of a compensating charge. Therefore, the solid solution reaction mechanism of in-situ-synthesized β-sialon in the Al_2_O_3_ matrix involved a double mechanism of unequivalence (or hetero-valence) and interstitial filling.

### 3.3. Strengthening and Toughening Mechanisms 

The main fracture mode of the ASN tool material was a mixed mode of inter-granular and transgranular fracture, as shown in the SEM micrograph on the fractured surface in Figure 6b. Typical characteristics of a transgranular fracture mode, such as cleavage steps (indicated by the white quadrate box in Figure 6b), were clearly observed, which indicated that ASN prepared at 1550 °C for 20 min had not only good interfacial bonding strength between the grains, but also high fracture energy dissipation in the process of crack propagation. As a result, the combination of transgranular fracture and intergranular fracture processes improved the strength and toughness of the ASN tool material. 

It can be seen in Figure 6b that a considerable number of grains were pulled out. When ASN fractured, the pullout holes of the grains (indicated by the white arrows), especially the elongated grains, indicated that the elongated β-sialon grains may be pulled out and ruptured. Due to the thermal expansion mismatch between Al_2_O_3_ (~8.0 × 10^−6^ K^−1^ at 0–1200 °C) and β-sialon (~3.2 × 10^−6^ K^−1^ at 0–1200 °C), the residual radial compressive stress was born on the elongated β-sialon, and a friction force was introduced on the β-sialon as the elongated β-sialon pulled out from the Al_2_O_3_ matrix. As a result, the frictional energy was consumed, and the fracture toughness of the composite was improved [42]. Furthermore, interface debonding between the elongated β-sialon and the Al_2_O_3_ matrix was observed from the cleavage planes of the elongated grain (denoted by the ellipse box), as shown in Figure 6b. Due to the thermal expansion mismatch between the elongated β-sialon and Al_2_O_3_, the internal stress developed during cooling with a compressive stress on the elongated β-sialon and a tension stress on the Al_2_O_3_ matrix. Thus, the shear stress was generated in the interface between the β-sialon and the Al_2_O_3_ matrix [10]. When the shear stress was higher than the Al_2_O_3_/β-sialon interfacial bonding strength, interface debonding occurred. Therefore, when the crack propagated to the elongated β-sialon, the crack propagation path was deflected by the elongated β-sialon and more fracture energy was dissipated, so the fracture toughness of the composite was improved. 

It can be seen from Figure 3b,d that, when crack propagation encountered the interlocking structure of β-sialon grains, it was beneficial to rapidly disperse and transfer the stress concentrated on the point or plane in the space range. The interlocking structure provided high elastic stiffness, high bearing capacity and impact resistance, and ultimately played a role in toughening and reinforcing the ASN ceramic tool materials. 

**Figure 9:**
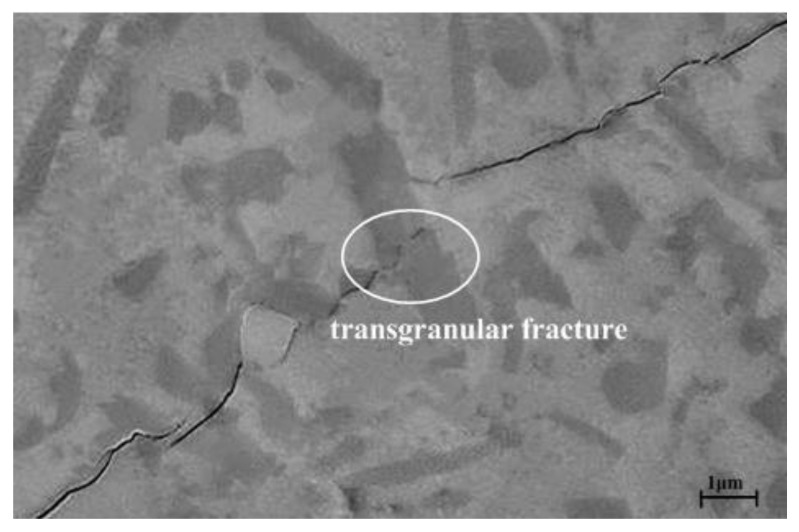
SEM micrograph on the polished surface of ASN and the crack propagation.

It can be seen from the intragranular microstructure shown in Figure 7a that, on the one hand, sub-interfaces occurred in the Al_2_O_3_ matrix grains, causing potential differentiation of the matrix grains and weakening the function of the main grain boundaries. On the other hand, due to the thermal expansion and elastic modulus mismatch between the β-sialon and the Al_2_O_3_ matrix, local tensile stress was generated around the β-sialon grains, and transgranular fracture occurred when the crack was propagated (denoted by the ellipse box, as shown in Figure 9). Transgranular fracture consumed the fracture energy and improved the fracture strength and toughness of the ASN ceramic tool material. At the same time, the compressive stress was generated on the grain boundary of the matrix. Once the main crack reached the grain boundary with compressive stress, the tensile stress of the main crack was partially or completely offset, which weakened the stress concentration at the tip of the main crack, and so the crack propagate would be pinned and terminated.

In addition, different from the interlocking and intragranular structures, during crack propagation, crack bridging may occur when the cracks encounter β-sialon grains with sub-micro- or nano-diameters dispersed in the matrix. If no cleavage occurs at the interface between the β-sialon grain and the matrix, the β-sialon grain would form a bridging zone at the crack tip and impose a stress causing the crack to close. If the bridged crack was to expand further, the interface between the β-sialon grain and the matrix must be dissociated, and, thus, the β-sialon grain of the bridging crack can be ruptured or be pulled out from the matrix. Finally, the fracture energy was consumed, and the fracture toughness of the ASN ceramic tool was improved.

Furthermore, the toughening mechanism of the β-sialon whiskers was basically similar to that of the elongated β-sialon grains. When the crack propagated under an external force, the β-sialon whiskers could also cause crack deflection and crack bridging. However, compared with the elongated grains, the whiskers were different in length, diameter, content, strength and the length of interfacial dissociation, so the degree of stress release of β-sialon whiskers on the crack tip was different from that of elongated β-sialon grains.

## 4. Conclusions

(1) A kind of multiscale β-sialon grain-reinforced Al_2_O_3_ matrix composite ceramic tool material was named ASN. In ASN, β-sialon (molecular formula: Si_4_Al_2_O_2_N_6_) was in situ synthesized by hot pressing and a solid solution reaction process. The solid solution reaction mechanism of β-sialon in the Al_2_O_3_ matrix was a double mechanism of unequivalence (or hetero-valence) and interstitial filling.
(2) By optimizing the sintering temperature and the holding time, the optimal comprehensive mechanical properties and higher relative density of the ASN tool material were obtained under a pressure of 32 MPa at 1550 °C for 20min. The flexural strength, fracture toughness, Vickers hardness and relative density were 997 ± 59 MPa, 6.4 ± 0.3 MPa·m^1/2^, 18.2 ± 0.4 GPa and 98.1 ± 0.2%, respectively.
(3) The multiscale β-sialon grains mainly consisted of elongated β-sialon grains with a diameter of 0.3–0.4 μm and an aspect ratio of 6–9, elongated β-sialon grains with a diameter of 70 nm and an aspect ratio of 10, β-sialon whiskers with a diameter of 0.2 μm and an aspect ratio of 12–15, and intragranular β-sialon whiskers with a diameter of 70 nm.
(4) Several strengthening and toughening mechanisms acted simultaneously in the composite ASN, such as mixed structure mode (intergranular and transgranular), elongated grain pullout, interface bonding, crack reflection, pinning and bridging.
(5) The nucleation mechanism of β-sialon and cutting experiments will be further investigated. The ASN ceramic cutting tool prepared in this study expanded the varieties of ceramic cutting tools and their application range and proportion in machining.

## Figures and Tables

**Table 1:** Comparison of process and room temperature mechanical properties of ASN and partial Al_2_O_3_-based ceramic tool materials.

Al_2_O_3_-Based Ceramic Tool	Main Ingredient	Flexural Strength (MPa)	Fracture Toughness (MPa·m^1/2^)	Vickers Hardness (GPa)	Relative Density (%)
ASN	Al_2_O_3_ + β-Sialon	997	6.4	18	98.1
LT55	Al_2_O_3_ + TiC	850	5.1	21	-
ATTC	Al_2_O_3μ_ + TiC_μ_ + TiC _n_ + Co	916	8.3	18	-
AWTC	Al_2_O_3μ_ + (W,Ti)C_μ_ + TiC_n_ + Co	882	7.2	19	-
WG-300	Al_2_O_3_ + SiC_W_	690	8.8	20	-

## Data Availability

The data presented in this study are available from the corresponding author upon reasonable request.
